# Clinical Trials and Treatment of ATL

**DOI:** 10.1155/2012/101754

**Published:** 2012-01-16

**Authors:** Kunihiro Tsukasaki, Kensei Tobinai

**Affiliations:** ^1^Department of Hematology, Atomic Bomb Disease Institute, Nagasaki University Graduate School of Biomedical Science, Nagasaki 852-8523, Japan; ^2^Hematology and Hematopoietic Stem Cell Transplantation Division, National Cancer Center Hospital, Tokyo 104-0045, Japan

## Abstract

ATL is a distinct peripheral T-lymphocytic malignancy associated with human T-cell lymphotropic virus type I (HTLV-1). The diversity in clinical features and prognosis of patients with this disease has led to its subtype-classification into four categories, acute, lymphoma, chronic, and smoldering types, defined by organ involvement, and LDH and calcium values. In case of acute, lymphoma, or unfavorable chronic subtypes (aggressive ATL), intensive chemotherapy like the LSG15 regimen (VCAP-AMP-VECP) is usually recommended if outside of clinical trials, based on the results of a phase 3 trial. In case of favorable chronic or smoldering ATL (indolent ATL), watchful waiting until disease progression has been recommended, although the long-term prognosis was inferior to those of, for instance, chronic lymphoid leukemia. Retrospective analysis suggested that the combination of interferon alpha and zidovudine was apparently promising for the treatment of ATL, especially for types with leukemic manifestation. Allogeneic hematopoietic stem cell transplantation (allo-HSCT) is also promising for the treatment of aggressive ATL possibly reflecting graft versus ATL effect. Several new agent trials for ATL are ongoing and in preparation, including a defucosylated humanized anti-CC chemokine receptor 4 monoclonal antibody, IL2-fused with diphtheria toxin, histone deacetylase inhibitors, a purine nucleoside phosphorylase inhibitor, a proteasome inhibitor, and lenalidomide.

## 1. Introduction

Adult T-cell leukemia-lymphoma (ATL) was first described in 1977 by Uchiyama et al. as a distinct clinico-pathological entity with a suspected viral etiology because of the clustering of the disease in the southwest region of Japan [1]. Subsequently, a novel RNA retrovirus, human T-cell leukemia/lymphotropic virus type I (HTLV-1), was isolated from a cell line established from leukemic cells of an ATL patient, and the finding of a clear association with ATL led to its inclusion among human carcinogenic pathogens [2–5]. In the mid-1980s and 1990s, several inflammatory diseases were reported to be associated with HTLV-1 [6–10]. At the same time, endemic areas for the virus and diseases have been found (reviewed in [11–13]). Diversity in ATL has been recognized and a classification of clinical subtypes of the disease was proposed [14]. This chapter will review the current recognition of ATL focusing on treatment of the disease. 

## 2. Clinical Features and Laboratory Findings of ATL

ATL patients show a variety of clinical manifestations because of various complications of organ involvement by ATL cells, opportunistic infections and/or hypercalcemia [11–14]. These three often contribute to the extremely high mortality of the disease. Lymph node, liver, spleen, and skin lesions are frequently observed. Though less frequently, digestive tract, lungs, central nervous system, bone, and/or other organs may be involved. Large nodules, plaques, ulcers, and erythroderma are common skin lesions [15–17]. Immune suppression is common. Approximately 26% of 854 patients with ATL had active infections at diagnosis in a prior nationwide study in Japan [14]. The incidence was highest in the chronic and smoldering types (36%) and lower in the acute (27%) and lymphoma types (11%). The infections were bacterial in 43%, fungal in 31%, protozoal in 18%, and viral in 8% of patients. The immunodeficiency at presentation in ATL patients can be exacerbated by cytotoxic chemotherapy. Individuals with indolent ATL might have no manifestation of the disease and are identified only by health checkups and laboratory examinations.

ATL cells are usually detected quite easily in the blood of affected individuals except for the smoldering type with mainly skin manifestations and lymphoma type [14]. These so-called “flower cells” have highly indented or lobulated nuclei with condensed chromatin, small or absent nucleoli, and a agranular and basophilic cytoplasm [18]. The histological analysis of aberrant cutaneous lesions or lymph nodes is essential for the diagnosis of the smoldering type with mainly skin manifestations and lymphoma type of ATL, respectively. Because ATL cells in the skin and lymph node can vary in size from small to large and in form from pleomorphic to anaplastic and Hodgkin-like cell with no specific histological pattern of involvement, differentiating between Sezary syndrome, other peripheral T-cell lymphomas and Hodgkin lymphoma versus ATL can at times be difficult without examinations for HTLV-1 serotype/genotype [13, 19].

Hypercalcemia is the most distinctive laboratory abnormality in ATL as compared to other lymphoid malignancies and is observed in 31% of patients (50% in acute type, 17% in lymphoma type, and 0% in the other two types) at onset [14]. Individuals with hypercalcemia do not usually have osteolytic bone lesions. Parathyroid hormone-related protein or receptor activator of nuclear factor kappa B ligand (RANKL) produced by ATL cells is considered the main factor causing hypercalcemia [20, 21].

Similar to serum LDH, *β*2-microglobulin, and serum thymidine kinase levels reflecting disease bulk/activity, the level of the soluble form of interleukin (IL)-2 receptor alpha-chain is elevated in the order of acute/lymphoma-type ATL, smoldering/chronic-type ATL, and HTLV-1 carriers as compared with normal individuals, perhaps with better accuracy than the other markers [22–24]. These serum markers are useful for detecting the acute transformation of indolent ATL as well as the early relapse of ATL after achieving responses by therapy.

Prototypical ATL cells have a mature alpha-beta T-cell phenotype, that is, they are terminal deoxynucleotidyl transferase- (TdT-)negative, cluster of differentiation (CD) 1a-negative, T-cell receptor alpha-beta positive, CD2-positive and CD5, CD45RO, and CD29-positive, and frequently do not express CD7 and CD26. A decline in the CD3 level with the appearance of CD25 indicates that the ATL cells are in an activated state. Most ATL cells are CD52-positive but some are negative, and this may correlate with the coexpression of CD30. About 90% of cases are CD4-positive and CD8-negative, and in rare cases either coexpress CD4 and CD8, are negative for both markers, or are only CD8-positive [25]. CC chemokine receptor 4 (CCR4) is expressed in more than 90% of cases and associated with a poor prognosis. Recent studies have suggested that the cells of some ATL may be the equivalent of regulatory T-cells because of the high frequency of expression of CD25/CCR4 and about half of FoxP3 [26–28].

## 3. Diagnosis of ATL

The diagnosis of typical ATL is not difficult and is based on clinical features, ATL cell morphology, mature helper-T-cell phenotype, and anti-HTLV-1 antibody in most cases [13]. Those rare cases, which might be difficult to diagnose, can be shown to have the monoclonal integration of HTLV-1 proviral DNA in the malignant cells as determined by Southern blotting. However, the monoclonal integration of HTLV-1 is also detected in some HAM/TSP patients and HTLV-1 carriers [29, 30]. After the diagnosis of ATL, subtype classification of the disease is necessary for the selection of appropriate treatment [14, 31].

## 4. Definition, Prognostic Factors, and Subtype Classification of ATL

ATL is a distinct peripheral T-lymphocytic malignancy associated with a retrovirus designated human T-cell leukemia virus type I or human T-cell lymphotropic virus type I (HTLV-1) [1, 11–14, 31].

Major prognostic indicators for ATL, which have been elucidated in 854 patients with ATL in Japan, the Lymphoma Study Group (LSG) of the Japan Clinical Oncology Group (JCOG) by multivariate analysis, were advanced performance status (PS), high lactic dehydrogenase (LDH) level, age of 40 years or more, more than 3 involved lesions, and hypercalcemia [32]. Also a classification of clinical subtypes into acute, lymphoma, chronic, and smoldering types was proposed based on prognostic factors and clinical features of the disease [14]. The leukemic subtypes include all of the chronic type and most of the acute and smoldering types. The acute type has a rapid course with leukemic manifestation (≥2% ATL cells) mostly, with or without lymphocytosis (>4 × 109/L) including ATL cells and most of the characteristic features of ATL-generalized lymphadenopathy, hepatosplenomegaly, skin involvement, other organ involvement, a high LDH value, and hypercalcemia. The symptoms and signs include abdominal pain, diarrhea, ascites, jaundice, unconsciousness, dyspnea, pleural effusion, cough, sputum, and chest X-ray abnormalities because of organ involvement, hypercalcemia, and/or opportunistic infections. The smoldering type shows an indolent course and 5% or more of leukemic cells in the peripheral blood without lymphocytosis but may include skin/lung involvement. The calcium level is less than the upper limit, and LDH level is less than 1.5 times the upper limit in smoldering ATL. The chronic type, with absolute lymphocytosis (4 × 109/L) less frequently showing flower cell morphology than the acute type, is frequently and occasionally associated with skin involvement and lymphadenopathy, respectively, and also usually shows a relatively indolent course. The calcium level is less than the upper limit, and the LDH level is less than double the upper limit of the chronic type. The lymphoma type presents with the manifestations of a nodal-lymphoma without leukemic cells, frequently with high LDH/Ca levels, a rapid course, and symptoms and signs similar to the acute type. In case of ATL, clinical subtype is more important than Ann Arbor stage for predicting prognosis and deciding treatment because of frequent leukemic manifestation defined as stage IV.

Additional factors associated with a poor prognosis include thrombocytopenia, eosinophilia, bone marrow involvement, a high interleukin (IL)-5 serum-level, C-C chemokine receptor 4 (CCR4) expression, lung resistance-related protein (LRP), p53 mutation, and p16 deletion by multivariate analysis [26, 27, 33–37]. Specific for the chronic type of ATL, high LDH, high blood urea nitrogen (BUN), and low albumin levels were identified as factors for a poor prognosis by multivariate analysis [11]. Primary cutaneous tumoral type although generally included among smoldering ATL had a poor prognosis in univariate analysis [15].

## 5. Clinical Course, Treatment, and Response Criteria of ATL

Treatment decisions should be based on the ATL subtype-classification and the prognostic factors at onset including those related with ATL and comorbidity [31]. As mentioned above, subtype-classification of this disease has been proposed based on the prognosis and clinical manifestations. Without treatment, most patients with acute-/lymphoma/type ATL die of the disease or infections within weeks or months. More than half of patients with smoldering ATL survive for more than 5 years without chemotherapy and transformation to aggressive ATL. Chronic ATL has the most diverse prognosis among the subtypes and could be divided into favorable and unfavorable by clinical parameters (serum albumin, BUN, and LDH levels) after a multivariate analysis [31].

Current treatment options for ATL include watchful waiting until the disease progresses, interferon alpha (IFN) and zidovudine (AZT) therapy, multiagent chemotherapy, allogeneic hematopoietic stem cell transplantation (allo-HSCT), and a new agent [15].

### 5.1. Watchful Waiting

At present, no standard treatment for ATL exists. Therefore, patients with the smoldering or favorable chronic type, who may survive one or more years without chemotherapy, excluding topical therapy for cutaneous lesions, should be observed and therapy should be delayed until progression of the disease [31]. However, it was recently found that the long-term prognosis of such patients was poorer than expected. In a long-term followup study for 78 patients with indolent ATL (favorable chronic- or smoldering-type) with a policy of watchful waiting until disease progression at a single institution, the median survival time was 5.3 years with no plateau in the survival curve. Twelve patients remained alive for >10 years, 32 progressed to acute ATL, and 51 died [38]. Recently, the striking benefit of early intervention to indolent ATL by IFN and an antiretroviral agent was reported by a meta-analysis [39]. This modality should be extensively evaluated by larger clinical trials to establish appropriate management practices for indolent ATL.

### 5.2. Chemotherapy

Since 1978, chemotherapy trials have been consecutively conducted for patients newly diagnosed with ATL by JCOG's Lymphoma Study Group (LSG) (Table 1) [40–45]. Between 1981 and 1983, JCOG conducted a phase III trial (JCOG8101) to evaluate LSG1-VEPA (vincristine, cyclophosphamide, prednisone, and doxorubicin) versus LSG2-VEPA-M (VEPA plus methotrexate (MTX)) for advanced non-Hodgkin lymphoma (NHL), including ATL [40, 41]. The complete response (CR) rate of LSG2-VEPA-M for ATL (37%) was higher than that of LSG1-VEPA (17%; *P* = .09). However, the CR rate was significantly lower for ATL than for B-cell NHL and peripheral T-cell lymphoma (PTCL) other than ATL (*P* < .001). The median survival time of the 54 patients with ATL was 6 months, and the estimated 4-year survival rate was 8%.

In 1987, JCOG initiated a multicenter phase II study (JCOG8701) of a multiagent combination chemotherapy (LSG4) for advanced aggressive NHL (including ATL). LSG4 consisted of three regimens: (1) VEPA-B (VEPA plus bleomycin), (2) M-FEPA (methotrexate, vindesine, cyclophosphamide, prednisone, and doxorubicin), and (3) VEPP-B, (vincristine, etoposide, procarbazine, prednisone, and bleomycin) [42]. The CR rate for ATL patients was improved from 28% (JCOG8101) to 43% (JCOG8701); however, the CR rate was significantly lower in ATL than in B-cell NHL and PTCL (*P* < .01). Patients with ATL still showed a poor prognosis, with a median survival time of 8 months and a 4-year survival rate of 12%.

The disappointing results with conventional chemotherapies have led to a search for new active agents. Multicenter phase I and II studies of pentostatin (2′-deoxycoformycin, an inhibitor of adenosine deaminase) were conducted against ATL in Japan [43]. The phase II study revealed a response rate of 32% (10 of 31) in cases of relapsed or refractory ATL (2CRs and 8PRs).

These encouraging results prompted the investigators to conduct a phase II trial (JCOG9109) with a pentostatin-containing combination (LSG11) as the initial chemotherapy [44]. Patients with aggressive ATL—that is, of the acute, lymphoma, or unfavorable chronic type—were eligible for this study. Unfavorable chronic-type ATL, defined as having at least 1 of 3 unfavorable prognostic factors (low serum albumin level, high LDH level, or high BUN), has an unfavorable prognosis similar to that for acute- and lymphoma-type ATL. A total of 62 untreated patients with aggressive ATL (34 acute, 21 lymphoma, and 7 unfavorable chronic type) were enrolled. A regimen of 1 mg/m^2^ vincristine on days 1 and 8, 40 mg/m^2^ doxorubicin on day 1, 100 mg/m^2^ etoposide on days 1 through 3, 40 mg/m^2^ prednisolone (PSL) on days 1 and 2, and 5 mg/m^2^ pentostatin on days 8, 15, and 22 was administered every 28 days for 10 cycles. Among the 61 patients evaluable for toxicity, four patients (7%) died of infections, two from septicemia, and two from cytomegalovirus pneumonia. Among the 60 eligible patients, there were 17CRs (28%) and 14 partial responses (PRs) (overall response rate [ORR] = 52%). The median survival time was 7.4 months, and the estimated 2-year survival rate was 17%. The prognosis in patients with ATL remained poor, even though they were treated with a pentostatin-containing combination chemotherapy. 

In 1994, JCOG initiated a phase II trial (JCOG9303) of an eight-drug regimen (LSG15) consisting of vincristine, cyclophosphamide, doxorubicin, prednisone, ranimustine, vindesine, etoposide, and carboplatin for untreated ATL [45]. Dose intensification was attempted with the prophylactic use of granulocyte colony-stimulating factor (G-CSF). In addition, non-cross-resistant agents, such as ranimustine and carboplatin, and intrathecal prophylaxis with MTX and PSL were incorporated. Ninety-six previously untreated patients with aggressive ATL were enrolled: 58 acute, 28 lymphoma, and 10 unfavorable chronic types. Approximately 81% of the 93 eligible patients responded (75/93), with 33 patients obtaining a CR (35%). The overall survival rate of the 93 patients at 2 years was estimated to be 31%, with a median survival time of 13 months. Grade 4 neutropenia and thrombocytopenia were observed in 65% and 53% of the patients, respectively, whereas grade 4 nonhematologic toxicity was observed in only one patient.

Dose intensification of CHOP with prophylactic use of G-CSF was expected to improve survival among patients with aggressive NHL, and our randomized phase II study (JCOG9505) comparing CHOP-14 (LSG19) and dose-escalated CHOP (LSG20) to treat aggressive NHL excluding ATL revealed biweekly CHOP to be more promising [46]. Therefore, we regarded biweekly CHOP as a standard treatment for NHL including aggressive ATL at the time of designing this phase III study.

To confirm whether the LSG15 regimen is a new standard for the treatment of aggressive ATL, JCOG conducted a phase III trial comparing modified (m)-LSG15 with biweekly CHOP (cyclophosphamide, hydroxy-doxorubicin, vincristine [Oncovin], and prednisone), both supported with G-CSF and intrathecal prophylaxis [47].

mLSG19, a modified version of LSG19, consisted of eight cycles of CHOP [CPA 750 mg/m^2^, ADM 50 mg/m^2^,VCR 1.4 mg/m^2^(maximum 2 mg) on day 1 and PSL 100 mg on days 1 to 5] every 2 weeks [46]. The modification was an intrathecal administration identical to that in mLSG15.

**Figure 1:**
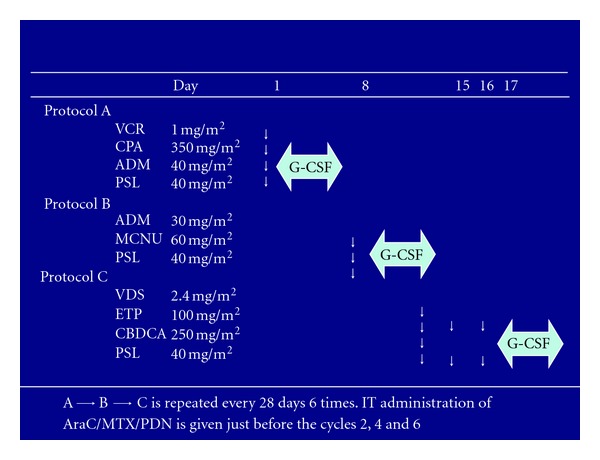
Regimen of VCAP-AMP-VECP in mLSG15. VCAP: vincristine (VCR), cyclophosphamide (CPA), doxorubicin (ADM), prednisone (PSL); AMP: ADM, ranimustine (MCNU), PSL; VECP: vindesine (VDS), etoposide (ETP), carboplatin (CBDCA), and PSL. ∗) MCNU and VDS are nitrosourea and vinca alkaloid, respectively, developed in Japan. A previous study on myeloma described that carmustine (BCNU), another nitrosourea, at 1 mg/kg is equivalent to MCNU at 0.8 to 1.0 mg/kg. VDS at 2.4 mg/m^2^ can be substituted for VCR, another vinca alkaloid used in this regimen, at 1 mg/m^2^ with possibly less myelosuppression and more peripheral neuropathy which can be managed by dose modification.

mLSG15 in JCOG9801 was a modified version of LSG15 in JCOG9303, consisting of three regimens: VCAP [VCR 1 mg/m^2^ (maximum 2 mg), CPA 350 mg/m^2^, ADM 40 mg/m^2^, PSL 40 mg/m^2^] on day 1, AMP [ADM 30 mg/m^2^, MCNU 60 mg/m^2^, PSL 40 mg/m^2^] on day 8, and VECP [VDS 2.4 mg/m^2^ on day 15, ETP 100 mg/m^2^ on days 15 to 17, CBDCA 250 mg/m^2^ on day15, PSL 40 mg/m^2^ on days 15 to 17] on days 15–17, and the next course was to be started on day 29 (Figure 1). The modifications in mLSG15 as compared to LSG15 were as follows: (1) The total number of cycles was reduced from 7 to 6 because of progressive cytopenia, especially thrombocytopenia, after repeating the LSG15 therapy. (2) Cytarabine 40 mg was used with MTX 15 mg and PSL 10 mg for prophylactic intrathecal administration, at the recovery phases of courses 1, 3, and 5 because of the high frequency of central nervous system relapse in the JCOG9303 study. Untreated patients with aggressive ATL were assigned to receive either six courses of mLSG15 every 4 weeks or eight courses of biweekly CHOP. The primary endpoint was overall survival. A total of 118 patients were enrolled. The CR rate was higher in the mLSG15 arm than in the biweekly CHOP arm (40% versus 25%, resp.; *P* = .020). As shown in Table 1, the median survival time and OS rate at 3 years were 12.7 months and 24% in the mLSG15 arm and 10.9 months and 13% in the biweekly CHOP arm [two-sided *P* = .169, and the hazard ratio was 0.75; 95% confidence interval (CI), 0.50 to 1.13]. A Cox regression analysis with performance status (PS 0 versus 1 versus 2–4) as the stratum for baseline hazard functions was performed to evaluate the effect on overall survival of age, B-symptoms, subtypes of ATL, LDH, BUN, bulky mass, and treatment arms. According to this analysis, the hazard ratio and two-sided P value for the treatment arms were 0.62 (95% CI, 0.38 to 1.01) and  .056, respectively. The difference between the crude analysis and this result was because of unbalanced prognostic factors, such as PS 0 versus 1, and the presence or absence of bulky lesions between the treatment arms. The progression-free survival rate at 1 year was 28% in the mLSG15 arm compared with 16% in the biweekly CHOP arm (two-sided *P* = .20).

 In mLSG15 versus mLSG19, rate of grade 4 neutropenia, grade 4 thrombocytopenia, and grade 3/4 infection were 98% versus 83%, 74% versus 17%, and 32% versus 15%, respectively. There were three toxic deaths in the former. Three treatment-related deaths (TRDs), two from sepsis and one from interstitial pneumonitis related to neutropenia, were reported in the mLSG15 arm. Two cases of myelodysplastic syndrome were reported, one each in both arms.

The longer survival at 3 years and higher CR rate with mLSG15 compared with mLSG19 suggest that mLSG15 is a more effective regimen at the expense of higher toxicity, providing the basis for future investigations in the treatment of ATL [47]. The superiority of VCAP-AMP-VECP in mLSG15 to biweekly CHOP in mLSG19 may be explained by the more prolonged, dose dense schedule of therapy in addition to 4 more drugs. In addition, agents such as carboplatin and ranimustine not affected by multidrug-resistance (MDR) related genes, which were frequently expressed in ATL cells at onset, were incorporated [48]. Intrathecal prophylaxis, which was incorporated in both arms of the phase III study, should be considered for patients with aggressive ATL even in the absence of clinical symptoms because a previous analysis revealed that more than half of relapses at new sites after chemotherapy occurred in the CNS [49]. However, the median survival time of 13 months in VCAP-AMP-VECP (LSG15/mLSG15) still compares unfavorably to other hematological malignancies, requiring further effort to improve the outcome.

### 5.3. Interferon-Alpha and Zidovudine

A small phase II trial in Japan of IFN alpha against relapsed/refractory ATL showed a response rate (all PR) of 33% (8/24), including 5 out of 9 (56%) chronic-type ATL [50]. In 1995, Gill and associates reported that 11 of 19 patients with acute- or lymphoma-type ATL showed major responses (5 CR and 6 PR) to a combination of interferon-alpha (IFN) and zidovudine (AZT) [51]. The efficacy of this combination was also observed by Hermine and associates; major objective responses were obtained in all five patients with ATL (four with acute type and one with smoldering type) [52]. Although these results are encouraging, the OS of previously untreated patients with ATL was relatively short (4.8 months) compared with the survival of those in the chemotherapy trials conducted by the JCOG-LSG (7 to 8 months) [53]. After that, numerous small phase II studies using AZT and IFN have shown responses in ATL patients [54–56]. High doses of both agents are recommended: 6–9 million units of IFN in combination with daily divided AZT doses of 800–1000 mg/day. Therapeutic effect of AZT and IFN is not through a direct cytotoxic effect of these drugs on the leukemic cells [57]. Enduring AZT treatment of ATL cell lines results in inhibition of telomerase which reprograms the cells to p53-dependent senescence [58].

Recently, the results of a “meta-analysis” on the use of IFN and AZT for ATL were reported [39]. A total of 100 patients received interferon-alpha and AZT as initial treatments. The ORR was 66%, with a 43% CR rate. In this worldwide retrospective analysis, the median survival time was 24 months and the 5-year survival rate was 50% for first-line IFN and AZT, versus 7 months and 20% for 84 patients who received first-line chemotherapy. The median survival time of patients with acute-type ATL treated with first-line IFN/AZT and chemotherapy was 12 and 9 months, respectively. Patients with lymphoma-type ATL did not benefit from this combination. In addition, first-line IFN/AZT therapy in chronic- and smoldering-type ATL resulted in a 100% survival rate at a median followup of 5 years. However, because of the retrospective nature of this meta-analysis based on medical records at each hospital, the decision process to select the therapeutic modality for each patient and the possibility of interference with OS by second-line treatment remains unknown. While the results for IFN/AZT in indolent ATL appear to be promising compared to those with watchful-waiting policy until disease progression, recently reported from Japan [38], the possibility of selection bias cannot be ruled out. A prospective multicenter phase III study evaluating the efficacy of IFN/AZT as compared to watchful-waiting for indolent ATL is to be initiated in Japan.

 Recently, a phase II study of the combination of arsenic trioxide, IFN, and AZT for chronic ATL revealed an impressive response rate and moderate toxicity [39]. Although the results appeared promising, the addition of arsenic trioxide to IFN/AZT, which might be sufficient for the treatment of chronic ATL as described above, caused more toxicity and should be evaluated with caution.

### 5.4. Allogeneic Hematopoietic Stem-Cell Transplantation (Allo-HSCT)

Allo-HSCT is now recommended for the treatment of young patients with aggressive ATL [31, 59]. Despite higher treatment-related mortality including graft versus host disease in a retrospective multicenter analysis of myeloablative allo-HSCT, the estimated 3-year OS of 33% is promising, possibly reflecting a graft versus ATL effect [60]. To evaluate the efficacy of allo-HSCT more accurately, especially in view of a comparison with intensive chemotherapy, a prospective multicenter phase II study of LSG15 chemotherapy followed by allo-HSCT is ongoing (JCOG0907).

Feasibility studies of allo-HSCT with reduced intensity conditioning for relatively aged patients with ATL also revealed promising results, and subsequent multicenter trials are being conducted in Japan [61, 62]. The minimal residual disease after allo-HSCT detected as HTLV-1 proviral load was much less than that after chemotherapy or AZT/IFN therapy, suggesting the presence of a graft-versus-ATL effect as well as graft-versus-HTLV-1 activity [61].

It remains unclear which type of allo-HSCT (myeloablative or reduced intensity conditioning) is more suitable for the treatment of ATL. Furthermore, selection criteria with respect to responses to previous treatments, sources of stem cells, and HTLV-1 viral status of the donor remain to be determined. Recently, a patient in whom ATL derived from donor cells developed four months after transplantation of stem cells from a sibling with HTLV-I was reported [63].

However, several other retrospective studies as well as those mentioned above on allo-HSCT showed a promising long-term survival rate of 20 to 40% with an apparent plateau phase despite significant treatment-related mortality.

### 5.5. Supportive Care

The prevention of opportunistic infections is essential in the management of ATL patients, nearly half of whom develop severe infections during chemotherapy. Some patients with indolent ATL develop infections during watchful waiting.

Sulfamethoxazole/trimethoprim and antifungal agents have been recommended as prophylaxes for Pneumocystis jiroveci pneumonia and fungal infections, respectively, in the JCOG trials [43–45]. While cytomegalovirus infections are not infrequent among ATL  patients, ganciclovir is not usually recommended as a prophylaxis [31]. In addition, in patients not receiving chemotherapy or allo-HSCT, antifungal prophylaxis may not be critical. An antistrongyloides agent, such as ivermectin or albendazole, should be considered to avoid systemic infections in patients with a history of exposure to the parasite in the tropics. Treatment with steroids and proton pump inhibitors may precipitate a fulminant strongyloides infestation and warrants testing before these agents are used in endemic areas [31]. Hypercalcemia associated with aggressive ATL can be corrected using chemotherapy in combination with hydration and bisphosphonate even when the performance status of the patient is poor.

### 5.6. Response Criteria

The complex nature of ATL, often with both leukemic and lymphomatous components, makes response assessment difficult. A modification of the JCOG response criteria was suggested by ATL consensus-meeting reflecting those for CLL and NHL which had been published later [31, 64, 65]. Recently, revised response criteria were proposed for lymphoma. New guidelines were presented incorporating positron emission tomography (PET), especially for the assessment of CR. It is well known and described in the criteria that several kinds of lymphoma including peripheral T-cell lymphomas were variably [18F] fluorodeoxyglucose (FDG) avid [66]. Meanwhile, PET or PET/CT is recommended for evaluations of response when the tumorous lesions are FDG-avid at diagnosis [31].

### 5.7. New Agents for ATL

#### 5.7.1. Purine Analogs

Several purine analogs have been evaluated for ATL. Among them, pentostatin (deoxycoformycin) has been most extensively evaluated as a single agent and in combination as described above [43, 46].

Other purine analogs clinically studied for ATL are fludarabine and cladribine. Fludarabine is among standard treatments for B-chronic lymphocytic leukemia and other lymphoid malignancies. In a phase I study of fludarabine in Japan, 5 ATL patients and 10 B-CLL patients with refractory or relapsed-disease were enrolled [67]. Six grade 3 nonhematological toxicities were only observed in the ATL patients. PR was achieved only in one of the 5 ATL patients and the duration was short. Cladribine is among standard treatments for hairy cell leukemia and other lymphoid malignancies. A phase II study of cladribine for relapsed/refractory aggressive-ATL in 15 patients revealed only one PR [68].

Forodesine, a purine nucleotide phosphorylase (PNP) inhibitor, is among purine nucleotide analogs. PNP is an enzyme in the purine salvage pathway that phosphorolysis 2′deoxyguanosine (dGuo). Purine nucleoside phosphorylase (PNP) deficiency in humans results in a severe combined immunodeficiency phenotype and the selective depletion of T cells associated with high plasma deoxyguanosine (dGuo) and high intracellular deoxyguanosine triphosphate levels in those cells with high deoxynucleoside kinase activity such as T cells, leading to cell death. Inhibitors of PNP, such as forodesine, mimic SCID in vitro and in vivo, suggesting a new targeting agent specific for T cell malignancies [69]. A dose escalating phase I study of forodesine is being conducted in Japan for T cell malignancies including ATL.

#### 5.7.2. Histone Deacetylase Inhibitor

Gene expression governed by epigenetic changes is crucial to the pathogenesis of cancer. Histone deacetylases (HDACs) are enzymes involved in the remodeling of chromatin and play a key role in the epigenetic regulation of gene expression. Deacetylase inhibitors (DACis) induce the hyperacetylation of nonhistone proteins as well as nucleosomal histones resulting in the expression of repressed genes involved in growth arrest, terminal differentiation, and/or apoptosis among cancer cells. Several classes of HDACi have been found to have potent anticancer effects in preclinical studies. HDACIs such as vorinostat (suberoylanilide hydroxamic acid: SAHA), romidepsin (depsipeptide), and panobinostat (LBH589) have also shown promise in preclinical and/or clinical studies against T-cell malignancies including ATL [70, 71]. Vorinostat and romidepsin have been approved for cutaneous T-cell lymphoma (CTCL) by the Food and Drug Administration in the USA. LBH589 has a significant anti-ATL effect in vitro and in mice [71]. However, a phase II study for CTCL and indolent ATL in Japan was terminated because of severe infections associated with the shrinkage of skin tumors and formation of ulcers in patients with ATL. Further study is required to evaluate the efficacy of HDACIs for PTCL/CTCL including ATL.

#### 5.7.3. Monoclonal Antibodies and Toxin Fusion Proteins

Monoclonal antibodies (MoAb) and toxin fusion proteins targeting several molecules expressed on the surface of ATL cells and other lymphoid malignant cells, such as CD25, CD2, CD52, and chemokine receptor 4 (CCR4), have shown promise in recent clinical trials.

Because most ATL cells express the alpha-chain of IL-2R (CD25), Waldmann et al. treated patients with ATL using monoclonal antibodies to CD25 [72]. Six (32%) of 19 patients treated with anti-Tac showed objective responses lasting from 9 weeks to longer than 3 years. One impediment to this approach is the quantity of soluble IL-2R shed by the tumor cells into the circulation. Another strategy for targeting IL-2R is conjugation with an immunotoxin (Pseudomonas exotoxin) or radioisotope (yttrium-90). Waldmann et al. developed a stable conjugate of anti-Tac with yttrium-90. Among the 16 patients with ATL who received 5- to 15-mCi doses, 9 (56%) showed objective responses. The response lasted longer than that obtained with unconjugated anti-Tac antibody [73, 74].

LMB-2, composed of the anti-CD25 murine MoAb fused to the truncated form of Pseudomonas toxin, was cytotoxic to CD25-expressing cells including ATL cells in vitro and in mice. Phase I/II trials of this agent showed some effect against hairy cell leukemia, CTCL, and ATL [6]. Six of 35 patients in the phase I study had significant levels of neutralizing antibodies after the first cycle. This drug deserves further clinical trials including in combination with cytotoxic agents.

Denileukin diftitox (DD; DAB(389)-interleukin-2 [IL-2]), an interleukin-2-diphtheria toxin fusion protein targeting IL-2 receptor-expressing malignant T lymphocytes, shows efficacy as a single agent against CTCL and peripheral T-cell lymphoma (PTCL) [75]. Also the combination of this agent with multiagent chemotherapy, CHOP, was promising for PTCL [76]. ATL cells frequently and highly express CD25 as described above, and several ATL cases successfully treated with this agent have been reported [77].

CD52 antigen is present on normal and pathologic B and T cells. In PTCL, however, CD52 expression varies among patients, with an overall expression rate lower than 50% in one study but not in another [78, 79]. ATL cells frequently express CD52 as compared to other PTCLs. The humanized anti-CD52 monoclonal antibody alemtuzumab is active against CLL and PTCL as a single agent. The combination of alemtuzumab with a standard-dose cyclophosphamide/doxorubicin/vincristine/prednisone (CHOP) regimen as a first-line treatment for 24 patients with PTCL showed promising results with CR in 17 (71%) patients; 1 had a partial remission, with an overall median duration of response of 11 months and was associated with mostly manageable infections but including CMV reactivation [80]. Major infections were Jacob-Creutzfeldt virus reactivation, pulmonary invasive aspergillosis, and staphylococcus sepsis.

ATL cells express CD52, the target of alemtuzumab, which was active in a preclinical model of ATL and toxic to p53-deficient cells, and several ATL cases successfully treated with this agent have been reported [81–83].

Siplizumab is a humanized MoAb targeting CD2 and showed efficacy in a murine ATL model. P1 dose-escalating study of this agent in 22 patients with several kinds of T/NK-cell malignancy revealed 6 responses (2 CR in LGL leukemia, 3 PR in ATL, and 1 PR in CTCL). However, 4 patients developed EBV-associated LPD [84]. The broad specificity of this agent may eliminate both CD4- and CD8-positive T cells as well as NK cells without effecting B cells and predispose individuals to the development of EBV lymphoproliferative syndrome.

CC chemokine receptor 4 (CCR4) is expressed on normal T helper type 27 and regulatory T (Treg) cells and on certain types of T-cell neoplasms [20, 21, 35]. KW-0761, a next generation humanized anti-CCR4 mAb, with a defucosylated Fc region, exerts strong antibody-dependent cellular cytotoxicity (ADCC) due to increased binding to the Fc*γ* receptor on effecter cells [85]. A phase I study of dose escalation with 4 weekly intravenous infusions of KW-0761 in 16 patients with relapsed CCR4-positive T cell malignancy (13 ATL and 3 PTCL) revealed that one patient, at the maximum dose (1.0 mg/kg), developed grade (G) 3 dose-limiting toxic effects, namely, skin rashes and febrile neutropenia and G4 neutropenia [86]. Other treatment-related G3-4 toxic effects were lymphopenia (*n* = 10), neutropenia (*n* = 3), leukopenia (*n* = 2), herpes zoster (*n* = 1), and acute infusion reaction/cytokine release syndrome (*n* = 1). Neither the frequency nor severity of these effects increased with dose escalation or the plasma concentration of the agent. The maximum tolerated dose was not reached. No patients had detectable levels of anti-KW-0761 antibody. Five patients (31%; 95% CI, 11% to 59%) achieved objective responses: 2 complete (0.1; 1.0 mg/kg) and 3 partial (0.01; 2 at 1.0 mg/kg) responses. Three out of 13 patients with ATL (31%) achieved a response (2 CR and 1 PR). Responses in each lesion were diverse, that is, good in PB (6 CR and 1 PR/7 evaluable cases), intermediate in skin (3 CR and 1 PR/8 evaluable cases), and poor in LN (1 CR and 2 PR/11 evaluable cases). KW-0761 was well tolerated at all the doses tested, demonstrating potential efficacy against relapsed CCR4-positive ATL or PTCL. Recently, results of subsequent phase II studies at the 1.0 mg/kg in relapsed ATL, showing 50% of response rate with acceptable toxicity profiles, reported [87]. A phase II trial of single agent KW-0761 at the 1.0 mg/kg in relapsed PTCL/CTCL and a phase II trial of VCAP-AMP-VECP combined with KW-0761 for untreated aggressive ATL are ongoing.

#### 5.7.4. Other Agents

A proteasome inhibitor, bortezomib (Velcade), and an immunomodulatory agent, lenalidomide (Revlimid), both have potent preclinical and clinical activity in T-cell malignancies including ATL, are now under clinical trials for relapsed ATL in Japan [88–90]. Other potential drugs for ATL include pralatrexate (Folotyn), a new agent with clinical activity in T-cell malignancies including ATL [91–93]. The agent is a novel antifolate with improved membrane transport and polyglutamylation in tumor cells and high affinity for the reduced folate carrier (RFC) highly expressed in malignant cells and has been approved by FDA recently for T-cell lymphoma including ATL.

### 5.8. Prevention

Two steps should be considered for the prevention of HTLV-1-associated ATL. The first is the prevention of HTLV-1 infections. This has been achieved in some endemic areas in Japan by screening for HTLV-1 among blood donors and asking mothers who are carriers to refrain from breast feeding. For several decades, before initiation of the interventions, the prevalence of HTLV-1 has declined drastically in endemic areas in Japan, probably because of birth cohort effects [94]. The elimination of HTLV-1 in endemic areas is now considered possible due to the natural decrease in the prevalence as well as the intervention of transmission through blood transfusion and breast feeding. The second step is the prevention of ATL among HTLV-1 carriers. This has not been achieved partly because only about 5% of HTLV-1 carriers develop the disease in their life time although several risk factors have been identified by a cohort study of HTLV-1 carriers (Joint Study of Predisposing Factors for ATL Development) [95]. Also, no agent has been found to be effective in preventing the development of ATL among HTLV-1 carriers.

## 6. Conclusions

Clinical trials have been paramount to the recent advances in ATL treatment, including assessments of chemotherapy, AZT/IFN, and allo-HSCT. Recently, a strategy for ATL treatment, stratified by subtype-classification, prognostic factors, and the response to initial treatment as well as response criteria, was proposed [31]. The recommended treatment algorithm for ATL is shown in Table 2. However, ATL still has a worse prognosis than the other T-cell malignancies [96].There is no plateau with an initial steep slope and subsequent gentle slope without a plateau in the survival curve for aggressive or indolent ATL treated by watchful waiting and with chemotherapy, respectively, although the prognosis is much better in the latter [38]. A prognostic model for each subgroup should be elucidated to properly identify the candidate for allo-HSCT which can achieve a cure of ATL despite considerable treatment-related mortality. Although several small phase II trials and a recent metaanalysis suggested IFN/AZT therapy to be promising, no confirmative phase III study has been conducted [39]. Furthermore, as described in the other chapters in detail, more than ten promising new agents for PTCL/CTCL including ATL are now in clinical trials or preparation. Future clinical trials on ATL as described above should be incorporated to ensure that the consensus is continually updated to establish evidence-based practical guidelines.

## Figures and Tables

**Table 1:** Results of sequential chemotherapeutic-trials of untreated patients with ATL (JCOG-LSG).

	J7801	J8101	J8701	J9109	J9303	JCOG9801	
	LSG1	LSG1/LSG2	LSG4	LSG11	LSG15	mLSG15/mLSG19	
Pts. No.	18	54	43	62	96	57	61
CR (%)	16.7	27.8	41.9	28.3	35.5	40.4	24.6
CR + PR (%)				51.6	80.6	72.0	65.6
MST (months)		7.5	8.0	7.4	13.0	12.7	10.9
2 yr. survival (%)				17.0	31.3		
3 yr. survival (%)				10.0	21.9	23.6	12.7
4 yr. survival (%)		8.0	11.6				

CR: complete remission, PR: partial remission, MST: median survival time.

**Table 2:** Strategy for the treatment of Adult T-Cell Leukemia-Lymphoma.

Smoldering- or favorable chronic-type ATL
(i) Consider inclusion in prospective clinical trials.
(ii) Symptomatic patients (skin lesions, opportunistic infections, etc.): Consider AZT/IFN or Watch and Wait.
(iii) Asymptomatic patients: Consider Watch and Wait.

Unfavorable chronic- or acute-type ATL
(i) If outside clinical trials, check prognostic factors (including clinical and molecular factors if possible):
(a) Good prognostic factors: consider chemotherapy (VCAP-AMP-VECP evaluated by a phase III trial against biweekly-CHOP) or AZT/IFN (evaluated by a meta-analysis on retrospective studies).
(b) Poor prognostic factors: consider chemotherapy followed by conventional or reduced intensity allo-HSCT (evaluated by retrospective and prospective Japanese analyses, resp.).
(c) Poor response to initial therapy: Consider conventional or reduced intensity allo-HSCT.

Lymphoma-type ATL
(i) If outside clinical trials, consider chemotherapy (VCAP-AMP-VECP).
(ii) Check prognostic factors (including clinical and molecular factors if possible) and response to chemotherapy:
(a) Good prognostic factors and good response to initial therapy: Consider chemotherapy followed by observation.
(b) Poor prognostic factors or poor response to initial therapy: Consider chemotherapy followed by conventional or reduced intensity allo-HSCT.
